# Implementing a model of integrated CKD management between primary and secondary care

**DOI:** 10.1093/ckj/sfaf335

**Published:** 2025-11-04

**Authors:** Philippa Jones, Hannah O’Keeffe, Rupert W Major, James Ritchie, Nil Sanganee, Smeeta Sinha, James O Burton

**Affiliations:** Clinical Pharmacy Service, General Practice Alliance, Northampton, UK; Donal O’Donoghue Renal Research Centre, Department of Renal Medicine, Northern Care Alliance NHS Foundation Trust, Salford, UK; Manchester Academic Health Sciences Centre, University of Manchester, Manchester, UK; Department of Population Health Sciences, University of Leicester, Leicester, UK; Department of Renal Medicine, University Hospitals of Leicester NHS Trust, Leicester, UK; Leicester, Leicestershire and Rutland Integrated Care Board, NHS England, Leicester, UK; Donal O’Donoghue Renal Research Centre, Department of Renal Medicine, Northern Care Alliance NHS Foundation Trust, Salford, UK; Leicester, Leicestershire and Rutland Integrated Care Board, NHS England, Leicester, UK; Donal O’Donoghue Renal Research Centre, Department of Renal Medicine, Northern Care Alliance NHS Foundation Trust, Salford, UK; Manchester Academic Health Sciences Centre, University of Manchester, Manchester, UK; Department of Renal Medicine, University Hospitals of Leicester NHS Trust, Leicester, UK; NIHR Leicester Biomedical Research Centre, University of Leicester, Leicester, UK; Leicester British Heart Foundation Centre of Research Excellence, University of Leicester, Leicester, UK

**Keywords:** chronic kidney disease, dashboards, integrated care, medicine optimization, risk stratification

## Abstract

Chronic kidney disease (CKD) is a common condition and important cardiovascular risk factor. However, CKD remains underdiagnosed and evidence-based medicines underutilized. In most healthcare systems, most CKD is managed in primary care. Optimal management in this setting can only be achieved with integration of care including early identification, prioritization, and use of the tools and skill mix available. This narrative review focuses on the importance of screening and identification in primary care, looking at innovative solutions and methods from other long-term conditions, particularly cardio–renal–metabolic conditions. Integrated care virtual multidisciplinary reviews, have demonstrated clinical and economic benefits, improved medication optimization, and reduced unnecessary referrals. However, implementation remains inconsistent, and prescribing of both established and novel therapies remains sub-optimal. Optimizing CKD care requires a system-wide approach that reinforces primary–secondary care collaboration, prioritizes early detection, and facilitates timely, evidence-based interventions. The inclusion of urine albumin: creatinine ratio testing, integrated digital tools, and shared accountability frameworks must be urgently adopted to realize improved outcomes and reduce the burden of CKD on individuals and healthcare systems alike.

## BACKGROUND

Chronic kidney disease (CKD) is a common condition affecting up to 10% of the adult population [1, 2]. It is set to become one of the top five non-communicable causes of death by 2040 [3, 4]. In May 2025 the World Health Assembly formally adopted a resolution to reduce the burden of kidney disease, marking international political recognition of the scale and gravity of CKD, and elevating it as a global priority [5]. It remains underdiagnosed and evidence-based medications remain underutilized. Most people living with CKD will not progress to end stage kidney disease (ESKD), but many will develop premature cardiovascular disease, live longer in ill health, and die prematurely.

The shift over the past 20 years for the NHS, and indeed most healthcare systems, has been for CKD and associated population management to primary care. This has not necessarily occurred with the associated movement of skills, resources and funding. In the UK, primary care is largely delivered through general practices that act as the first point of contact for most patients, responsible for population-level management of long-term conditions including CKD. Increasingly, however, aspects of long-term condition care are being delivered at neighbourhood and ‘super-neighbourhood’ levels through Primary Care Networks and Integrated Care Systems. This transition reflects recognition that traditional practice-level delivery is not optimized for population health management. Unsurprisingly, this unplanned shift has led to underutilization of evidence-based interventions for most people living with kidney disease. Variability in practice-level performance is a particular challenge in the UK system, but this is also a relevant lesson internationally: population health approaches to CKD management must address local inequities in workforce, infrastructure, and capability.

Optimal care can only be achieved through truly integrated care between primary and secondary care to support the earlier identification of non-cardiometabolic related CKD where risk of rapid progression ESKD is higher, while supporting primary care services to deliver management at a population level. This has the potential to reduce the excess cardiovascular disease and ESKD associated with late diagnosis and sub-optimal management.

In this narrative review (including opinion based on the experiences of the authors), written from a secondary care perspective, we aim to identify potential barriers and facilitators to truly integrated care, including: the monitoring and identification of CKD in a systematic way, use of population health management tools such as dashboards, integrated care models to optimize care, risk stratification through the Kidney Failure Risk Equation (KFRE), and proteinuria testing.

Many of the examples we draw on are UK-based [e.g. Quality and Outcomes Framework (QOF) incentivization, National Institute for Health and Care Excellence (NICE) guidance, Additional Reimbursable Roles Scheme, and the Leicester LUCID programme] and specific to the NHS context, reflecting both the opportunities and challenges of a nationally organized system. However, the principles underpinning these approaches—such as incentivization of early CKD detection, use of risk stratification tools to prioritize specialist input, and embedding primary–secondary care collaboration—are potentially transferable to other health systems. To support generalizability, we draw parallels to programmes in other countries (e.g. home-based albuminuria screening in the Netherlands, dashboard-driven diabetes care in the EU, virtual nephrology care in the USA and Australia), and identify shared elements that may be adapted across different health system structures.

### Screening and identification of CKD in primary care

CKD is predominantly identified and managed in primary care, and early identification relies on the appropriate screening of high-risk individuals. Current recommendations are that all patients with risk factors, including diabetes, hypertension, and cardiovascular disease, should have screening for CKD with both eGFR and urine albumin:creatinine ratio (uACR) [6, 7]. Increasingly, the management of long-term conditions is becoming intertwined, particularly across the domains of cardio-renal-metabolism (CRM), due to the strong interaction of these conditions and an evolving convergence on similar pillars of treatment [8–11]. CKD is an important mediator of cardiovascular risk, and patients are far more likely to die from cardiovascular disease than reach ESKD [12, 13]. Early identification of CKD enables proactive management of cardiovascular risk factors at the same time as employing strategies that delay CKD progression.

Unfortunately, late presentation rates of ESKD remain high both in the UK and globally [14–16]. Late presentations are typically defined as patients first presenting to renal specialist care <3 months before they require kidney replacement therapy (KRT). In the UK in 2022, 18.6% of KRT starts were late presenters [16]. This denies these patients an opportunity for treatments to slow CKD progression, and also means they are less likely to be pre-emptively listed for transplant or start KRT with definitive access via a fistula or graft, conferring overall poorer outcomes. As well as helping to reduce population risk for CKD progression and improving cardiovascular outcomes, a focus on earlier identification and risk stratification at a primary care level may also serve to reduce late presentation rates.

Awareness and recognition of CKD in primary care have historically been poor. The national prevalence of coded CKD (stages 3a–5) in England is currently 4% [17]; significantly below an estimated actual prevalence of 10% [1, 2, 18]. This is, in part, linked to frequency of ACR testing, a critical element in the diagnosis of CKD.

#### The importance of ACR testing

Data from the DEMAND study, a global, cross-sectional study evaluating >30 000 people living with type 2 diabetes revealed that 22% of that referred cohort met a diagnosis of CKD based on an eGFR of <60 ml/min/1.73 m^2^ [19]. However, when measures of albuminuria were factored in, the total number of people meeting the diagnostic criteria for CKD rose to 58%, highlighting the critical importance of ACR testing in the early identification of people with CKD, within the primary care setting.

**Figure 1:**
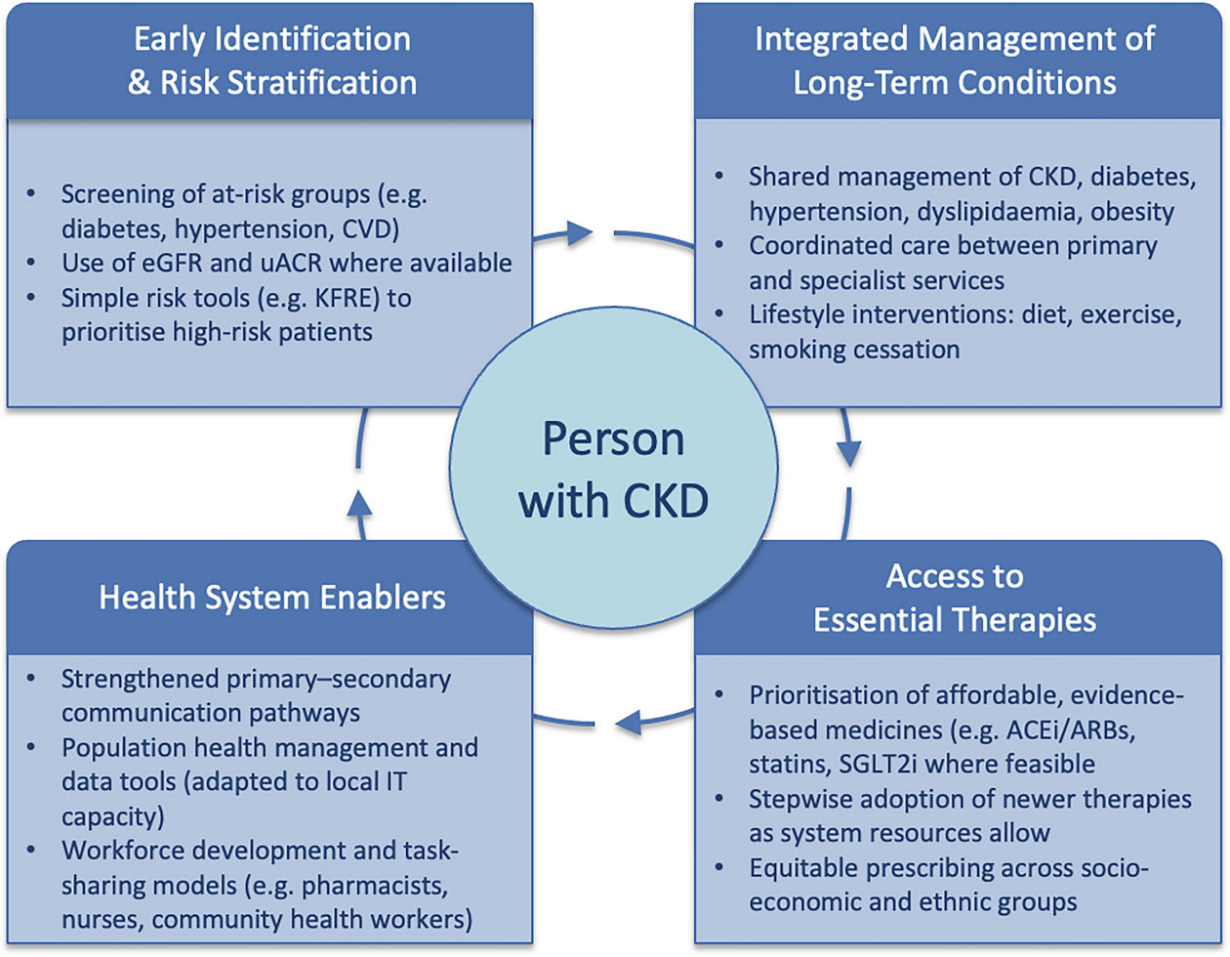
Principles of integrated CKD care that can be applied across diverse health systems. The underlying principles are generalizable: early identification, integration with long-term condition management, equitable access to essential therapies, and enabling system infrastructure. Adaptation to LMICs should prioritize affordable, accessible interventions, and workforce models that align with local health system capacity.

Although international studies such as DEMAND have demonstrated the importance of albuminuria testing, such cohorts disproportionately represent high-income countries. Globally, most people with CKD do not have access to KRT if they progress to kidney failure, and in many low- and middle-income countries’ (LMICs) access to therapies that best protect against cardiovascular and kidney outcomes remains extremely limited. To avoid overgeneralization, we propose that the transferable element is not the precise structure of UK or other programmes but rather the core principles of integrated CKD care: early identification, cardiovascular risk reduction, medicines optimization, and coordinated primary–specialist communication. Figure 1 outlines how these principles may be adapted for LMICs as well as high-income settings.

**Figure 2:**
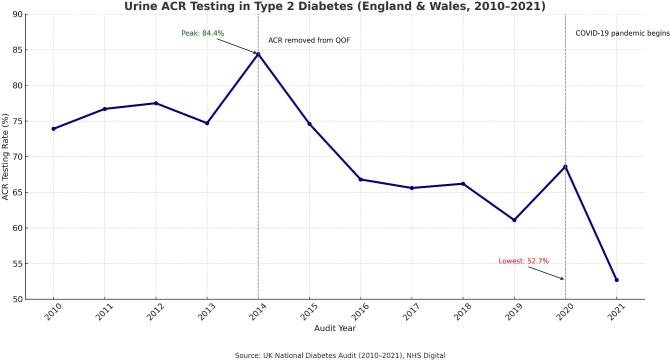
The annual ACR testing rate of people living with diabetes in the UK from 2010 to 2021. The dotted lines represent the impact of: the removal of financial incentives (QOF) for ACR testing in primary care in 2014 and; the COVID-19 pandemic (2020). Data retrieved from the UK National Diabetes Audit [72].

It is important to distinguish between different types of national policy levers within the UK. The National Institute of Health and Social Care Excellence (NICE) produces both clinical guidelines (advisory in nature, with variable uptake) and Technology Appraisals (mandatory where approved). By contrast, commissioning frameworks such as the QOF directly link financial incentives to delivery of defined indicators, creating stronger levers for behaviour change in clinical practice. This distinction helps explain differences in adoption and consistency of CKD care across the NHS. For example, ACR testing was financially incentivized under the QOF initiative, which up to 2014 included ‘The percentage of patients on the CKD register whose notes have a record of a urine albumin:creatinine ratio (or protein:creatinine ratio) test in the preceding 12 months’ [20]. According to the UK National Diabetes Audit, annual ACR testing rates in people living with diabetes reached a peak of 84.4% in 2014, at which point the incentives were withdrawn, resulting in a subsequent decline to 52.7% in 2021 (see Fig. 2), which has remained low [21]. The figures are worse for people with CKD caused by aetiologies other than diabetes in which awareness is even lower. As of June 2024, only 44% of people nationally with known CKD had a uACR in the preceding 12 months and just 29% of those with hypertension [17, 22] leading to calls for urgent action to reinstate incentives within primary care [23]. The removal of ACR testing as a system priority had additional consequences. This change potentially led to a perception among healthcare providers that ACR testing is less valuable compared with other assessments such as estimated glomerular filtration rate (eGFR), further contributing to reduced testing rates across NHS England [24].

Despite the decline in testing rates, ACR testing remains a vital tool for assessing kidney function and identifying early signs of kidney damage, especially in patients with diabetes. Early detection through ACR testing allows for timely interventions that can slow disease progression and reduce the risk of cardiovascular complications; the REVEAL-CKD Study demonstrated that a delayed diagnosis of CKD by 1 year is associated with a 40% increased risk of progressive CKD, a 63% increased risk of kidney failure, and an 8% increased risk of major adverse cardiovascular events [25].

### Recommendations for commissioners of primary care providers

#### Reinforce the importance of ACR testing

Healthcare providers should continue to prioritize ACR testing in people with established risk factors for CKD, recognizing its role in early CKD detection. For example, by consideration of inclusion within local incentive schemes.

#### Educate patients

A recent survey of people with kidney disease showed that almost two-thirds of people with both diabetes and hypertension were not told they were at increased risk of CKD, despite a wish to know, thereby missing opportunities to improve outcomes by providing tailored CKD information and support [26]. Informing patients about the significance of ACR testing in diagnosing and monitoring kidney disease (and cardiovascular risk) activates patients and encourages partnership in both guideline recommended diagnostic pathways and management.

#### Use alternative testing methods

Healthcare providers should consider implementing home-based ACR testing solutions, which have been found to be feasible and acceptable, potentially improving testing rates among high-risk populations [27].

### Innovative approaches to facilitate earlier detection

As described before, the health system prioritization of CKD and financial incentivization are important, however, moving beyond a simple process metric such as uACR testing and fostering support across the whole CKD pathway is vital to reduce the excess risk associated with CKD. Barriers to optimal CKD screening and diagnosis are present at patient, clinician ,and system levels as illustrated in Table 1.

**Table 1:** The barriers to CKD screening and diagnosis across patient, clinician, and system levels.

Level	Barriers
Patient [73–75]	- Lack of awareness of CKD or its risks
	- Absence of symptoms in early stages
	- Limited health literacy or understanding of test results
	- Socio-economic barriers (e.g. transport, cost, competing priorities)
	- Cultural or language barriers
	- Fear or denial of diagnosis
Clinician [23, 76, 77]	- Underestimation of CKD importance in asymptomatic patients
	- Limited time in consultations
	- Confusion over guidelines (e.g. ACR vs. eGFR thresholds)
	- Inadequate training in interpreting CKD labs
	- Therapeutic inertia (delayed response to abnormal results)
	- Discontinuation of incentivized testing (e.g. removal of QOF indicators)
System [78]	- Fragmented care between primary and secondary services leading to inequitable healthcare funding models (both between and within countries)
	- Lack of integrated or accessible patient records
	- Insufficient IT prompts/reminders for CKD screening
	- Inconsistent test coding or data recording
	- Resource limitations (e.g. lab access), workforce shortages and uneven distribution of specialist and primary care staff
	- Focus on short-term outcomes over long-term disease prevention

An innovative example to address screening challenges is from the Dutch ‘Check@Home’ consortium which aims to screen patients for cardiovascular disease, kidney disease, and diabetes at home [28]. This builds on the THOMAS study, which was a prospective trial, also conducted in the Netherlands, that demonstrated high participation, high identification of albuminuria using at-home testing, and was shown to be cost effective [29, 30]. In the UK, there has been an increasing focus on integrated CKD care between primary and specialist care in recent years. This has been advocated for by the Renal Service Transformation Programme, with a strong emphasis on case finding, risk stratification and integrated clinics and dashboards [31, 32]. A notable example of such integrated care work is via the LUCID programme [33].

Future optimal CKD care needs to focus on early identification and management with an integrated (both across CRM and between primary and specialist care) patient-centred approach. Consideration should be given to inclusive approaches such as at-home point of care testing and coordinated CKD screening at appointments for other chronic diseases. Early identification and proactive management across the population has the potential to reduce the numbers of patients reaching ESKD, reduce late presentations of CKD, and reduce the cardiovascular burden for those with CKD.

### Use of dashboards

Actionable data visualization in the form of dashboards should be considered when establishing integrated CKD services. Healthcare dashboards provide ‘snapshots’ of key metrics for clinical management, population health or quality improvement [34]. Dashboards should be co-developed with clinicians and patients, and focus on improving care across the CKD pathway, as well as reducing health inequalities and unwarranted variation. In addition, dashboards should undergo evaluation and iteration over time to ensure they are meeting the objectives for which they were designed and implemented.

There is evidence that implementation of dashboards for integrated chronic disease management can improve adherence to guidelines and improve patient outcomes with multiple examples across different chronic disease areas [34, 35]. A European Union project found successful implementation of a diabetes dashboard with integrated clinical decision support and resulted in improved screening for diabetes complications [36]. Another Diabetes study demonstrated how a dashboard supported integrated care in the UK including with improvement seen in key treatment targets, as well as the data being utilized to guide discussions at multidisciplinary team meetings [37]. A study on the use of a dashboard for hepatitis C care in the Veterans Affairs healthcare system found rapid uptake and high user satisfaction [38]. Multiple examples of dashboards enhancing population health in lipid management were highlighted in a recent scoping review [39]. A pilot study of an integrated CKD dashboard highlighted the potential to improve capacity in specialist renal care, improve evidence-based treatment, and reduce variation [31]. At the per-patient level, effective dashboards can mitigate information overload and increase clinician efficiency [40, 41]. As integrated CKD services aim to have impact at a person and population level, it is important that both drivers are considered during design and development to consider the impact of presented information on user actions, such as task performance and workflow interaction while maximizing potential utility [41].

**Figure 3:**
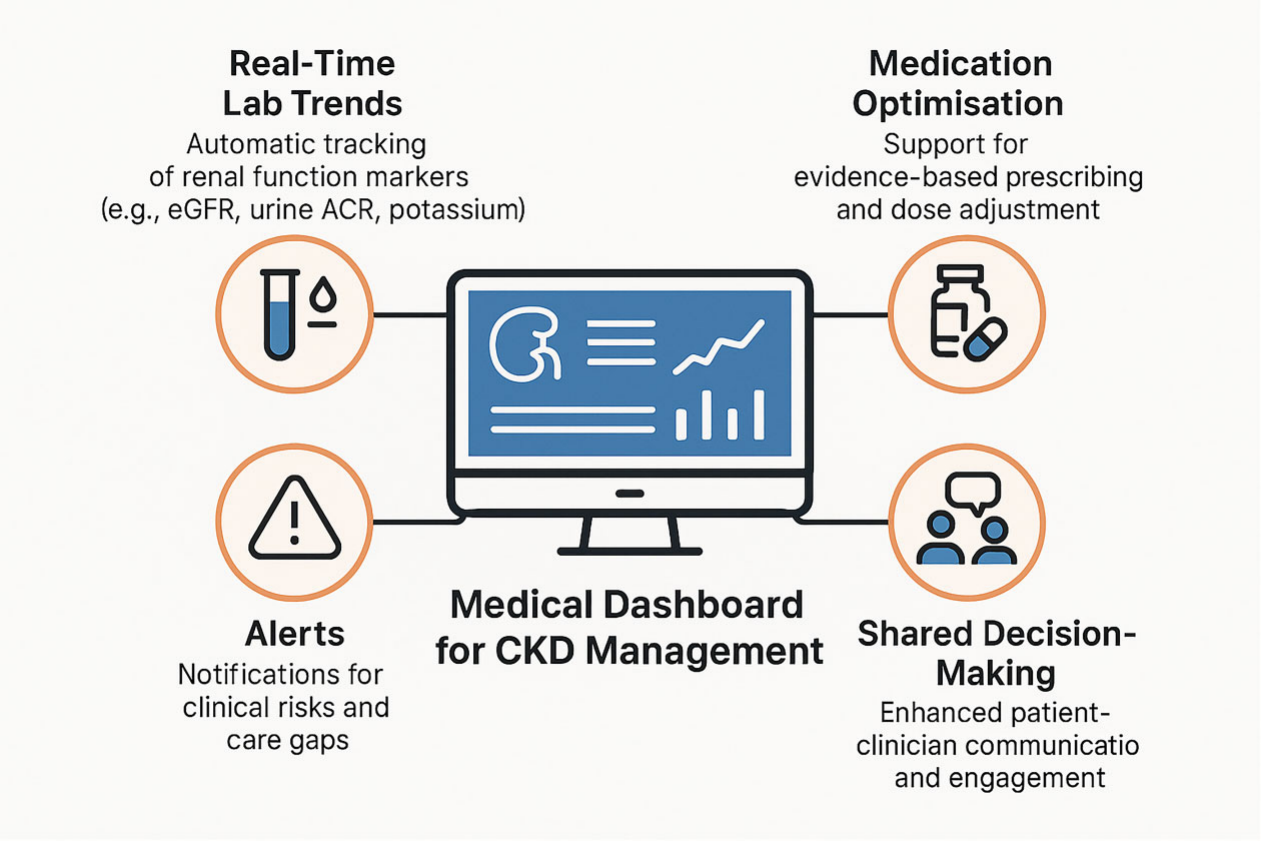
Key benefits of a digital medical dashboard to support management of people with CKD. (**a**) case finding through real-time laboratory trends, (**b**) medicines optimization (including flagging concerns about contra-indications or drug interactions), (**c**) alerts to reduce therapeutic inertia and prevent adverse events, and (**d**) shared decision making both for the patient at the centre and between clinicians.

The use of dashboards in integrated CKD care has the potential to improve early identification, risk stratification, patient prioritization, medication optimization, and monitoring of progress over time. CKD is perhaps uniquely suited to the use of such dashboards, with diagnosis and staging based on biochemical parameters (eGFR and uACR) and the availability of a validated risk stratification tool in the KFRE [6, 41–43]. The use of such dashboards may also facilitate data-driven, continuous improvement of processes and services. Figure 3 illustrates some of the key benefits of a digital medical dashboard designed to support the management of individuals with CKD, including case finding through real-time laboratory trends, medicine optimization (including flagging concerns about contra-indications or drug interactions), alerts to reduce therapeutic inertia and prevent adverse events, and shared decision making both for the patient at the centre and between clinicians.

However, successful implementation of dashboards and virtual care models is contingent on adequate IT infrastructure, interoperability between electronic systems, and equitable access to digital devices. Without these, dashboards risk reinforcing existing inequities and digital exclusion. Programmes must therefore incorporate safeguards, such as simplified user interfaces, patient access alternatives, and proactive monitoring of disparities in uptake and benefit.

### Opportunities to optimize care through enhanced primary–secondary care collaboration

Recent strategies have demonstrated that shifting long-term condition management from hospitals to primary care improves patient outcomes [44]. Evidence has long established that primary care is pivotal in the management of long-term conditions [45] and that effective CKD management relies on coordinated care between primary and secondary services. Although fragmentation and inconsistent communication between these levels of care remain key barriers to optimal therapy, inequities in CKD care extend beyond clinical practice variation.

**Figure 4:**
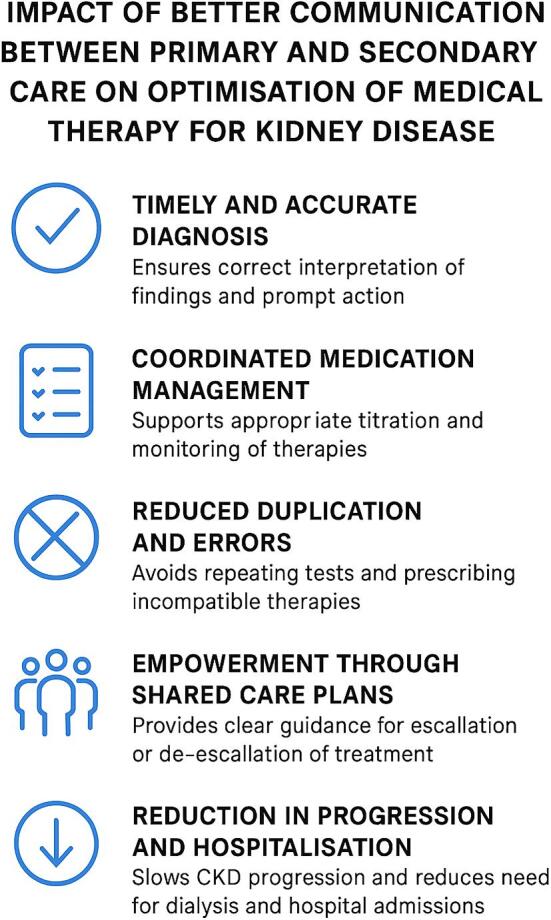
The impact of improved communication between primary and secondary care on the optimization of medica therapy for CKD.

Primary care practitioners are responsible for early CKD management, but interpretation of results, especially in the context of comorbidities and fluctuating renal function, often requires specialist oversight. Improved communication facilitates timely nephrology input through expedited referrals, electronic consultations, or shared access to laboratory systems (see Fig. 4).

There is strong clinical evidence base supporting the management of CKD to improve patient outcomes. Established management options such as angiotensin converting enzyme inhibitors and angiotensin receptor blockers remain first line [46], with newer therapies such as SGLT2i [47, 48], and non-steroidal mineralocorticoid receptor antagonists such as finerenone [49], both offering additional clinical benefits in specific patient groups. Nevertheless, despite the well-established cardiovascular and kidney benefits of SGLT2i in people CKD, their use has still not been successfully implemented into clinical practice. A recent study by *Forbes et al.* of >6.5 m adults with CKD in the UK demonstrated that SGLT2i were prescribed to just 17.0% of those with a guideline directed indication for treatment [50]. Of concern was the fact that individuals of Black ethnicity (OR 0.84, *P *< .0001) and those of lower socio-economic status (OR 0.72, *P *< .0001) were less likely to be prescribed an SGLT2 inhibitor. A similar picture can be seen in the USA where prescribing of evidence-based therapies such as SGLT2 inhibitors is lower among individuals from socio-economically deprived backgrounds and those of Black ethnicity [51], highlighting the intersection of biological risk and systemic inequality. Structural inequities in healthcare funding models and workforce availability further compound these disparities. Addressing CKD care therefore requires not only integration across primary and secondary care (enabling specialist expertise to be implemented earlier in the patient journey to improve outcomes) but also explicit attention to equity in implementation.

NICE guidance on Finerenone in the UK, published in 2023, suggests a transition to primary care prescribing as experience of the medicine grows [52], yet open-source data suggest prescribing rates remain low [53]. Improved integration of care will accelerate the sharing of clinical experience from specialists in secondary care to primary care prescribers. With growing evidence, such as the FLOW trial, highlighting the benefits of GLP-1 receptor antagonists in reducing major kidney events and major cardiovascular events [54], ensuring the rapid implementation of new therapies in an efficient and safe way will remain a pressing concern. As an example, current data indicate the roll out of tirzepatide may take 12 years in England [55] and thresholds for treatment within guidelines (in this case BMI) often do not match the inclusion criteria that are applied in clinical trials of GLP-1 agonists for CKD, creating a disconnect between trial evidence and current guidelines for implementation.

The Additional Reimbursable Roles Scheme in primary care has expanded the multidisciplinary support available in primary care. Roles such as pharmacists and health and wellbeing coaches can be usd to enhance the management of patients with CKD. Health and Wellbeing coaches are ideally placed to promote the essential lifestyle advice that is crucial to slowing CKD progression. Pharmacist-led medication management has been shown to improve outcomes for patients with chronic diseases, particularly around cardiovascular risk factors [56].

There is a limited but growing body of evidence supporting virtual care for CKD. East London have been at the forefront in the UK; demonstrating and sustaining benefits of virtual care [57, 58]. Over 3 years, most of their patients were managed virtually (>80%) without the need for a traditional outpatient review, and there was a reduction in waiting times for specialist input from 64 to 6 days [58]. Rapid uptake was seen (to 166 GP practices), with GP acceptability and a reduction in both new and follow-up outpatient appointments.

An example from diabetes care is a prospective trial randomizing patients to virtual or traditional care [59]. Meaningful glycaemic control improvements were seen in both arms. Notably, patients in the virtual group had an increased number of treatment adjustments and were more likely to transition back to primary care.

Other examples in the CKD literature include transition of low-risk patients already within secondary care to virtual care thus increasing capacity for complex cases, review of new low-risk referrals, and review of high-risk patients in primary care [60–62]. A study reporting the assessment of low-risk referrals via a virtual model found significant reductions in time to specialist review (48 hours versus 4 weeks) and cost savings of £111.56 per patient [61]. The PSP-CKD study, a cluster randomized controlled trial demonstrated that specialist nurse-led CKD care could improve coding, proteinuria measurement, and blood pressure in primary care [63].

K-CHAMP was a cluster randomized trial comparing an electronic record-based intervention with usual care [64–66]. The intervention bundle included nephrology guidance through e-consults, pharmacist-led medication management, and patient education. There was no difference in progression of CKD compared to usual care, and SGLT2i and GLP-1 use were higher in the virtual arm [64, 66].

The recently published LUCID project reports the feasibility of integrated CKD care at scale, demonstrating both clinical and economic acceptability of such a strategy [33]. In this programme most patients (54.5%) had CKD medication optimization as a result of virtual CKD multidisciplinary team review and led to a reduction in referrals and expedited specialist clinic review where indicated.

Most individuals with CKD live with multiple long-term conditions, most commonly type 2 diabetes, hypertension, and cardiovascular disease. In many cases, these long-term conditions are causal for CKD. Much of the management therefore overlaps, focusing on shared drivers such as blood pressure control, weight management, and lipid lowering. Despite this, the evidence base for integrated care models spanning multiple long-term conditions is limited, and CKD is often managed in isolation. In the same way as the LUCID project, future work should continue prioritize evaluation of multimorbidity-focused models of care that align interventions across cardio–renal–metabolic domains.

By risk stratifying patients, virtual care can increase capacity to see complex and high-risk patients in a timely fashion, while lower risk CKD patients in primary care can have improved management and continuity, with virtual specialist input. The studies so far suggest virtual care is an acceptable and economically viable model for managing CKD.

### Use of the KFRE to streamline health resource utilization

The use of risk prediction tools is now established in national and international guidelines for supporting patient care regarding the risk of progression to KRT. The KFRE has undergone extensive development and external evaluation across multiple countries [42, 43]. Its use has the potential to optimize healthcare delivery and resource allocation, however, implementation into routine clinical care has been slow despite the limited amount of information required to calculate it (between four and eight variables) and the relative simplicity of its algorithm compared with more complex data-driven machine learning techniques [67]. Measurement of urine ACR remains a major implementation barrier for KFRE.

Use of KFRE may have multiple benefits to individual and population-based care through early referral for those at higher risk, while reducing unnecessary referrals, provide earlier identification for medicines optimization for higher risk patients and improving population risk stratification through interventions such as dashboards to plan expected demands for future KRT services.

Analysis by Major *et al.* and repeated by NICE as part of their appraisal of KFRE suggested that its use instead of a referral threshold of eGFR<30 ml/min/1.73 m^2^ (stage G4 CKD) would reduce referral eligibility by at least 5% while identifying more people who would progress to need KRT [43]. KFRE’s role has also been studied to identify when to include multidisciplinary care and for timing of fistula formation [68–71]. In addition to potentially reducing unnecessary referral and planning more complex care, KFRE may aid with assisting targeted interventions by identifying those at highest risk for earlier medicines optimization.

### Conclusion and a call for action

CKD has been transformed in the last 20 years, through its diagnosis, staging, management and recognition as a major contributor to premature population ill health. As a community, we now face the problem of implementation of these research developments into routine care. Prescribing for angiotensin converting enzyme inhibitors and angiotensin receptor blockers remain poor despite 20 plus years of use in primary and secondary care; lessons regarding sub-optimal prescribing of guideline directed medical therapies have not been learned nor applied to newer agents such as SGLT2i. Challenges remain to improving rates of uACR testing with the withdrawal of financial incentivization having led to a precipitous drop in England for annual testing.

We must not lose this generational opportunity to improve the care for people living with CKD and other long-term conditions. Robust communication between primary and secondary care is foundational to the optimization of medical therapy for CKD. It enables earlier diagnosis, safer prescribing, shared responsibility, and more efficient use of healthcare resources. As the prevalence of CKD rises, embedding integrated communication pathways and shared care models between primary and secondary care is essential to improving clinical outcomes and quality of life for people living with kidney disease.

## Data Availability

No new data were generated or analysed in support of this research.

## References

[1] Hill NR, Fatoba ST, Oke JL et al. Global prevalence of chronic kidney disease–a systematic review and meta-analysis. PLoS ONE 2016;11:e0158765. 10.1371/journal.pone.0158765 27383068 PMC4934905

[2] Jager KJ, Kovesdy C, Langham R et al. A single number for advocacy and communication—worldwide more than 850 million individuals have kidney diseases. Nephrol Dial Transplant 2019;34:1803–5. 10.1093/ndt/gfz174 31566230

[3] Foreman KJ, Marquez N, Dolgert A et al. Forecasting life expectancy, years of life lost, and all-cause and cause-specific mortality for 250 causes of death: reference and alternative scenarios for 2016-40 for 195 countries and territories. Lancet 2018;392:2052–90. 10.1016/S0140-6736(18)31694-5 30340847 PMC6227505

[4] World Health Organisation. Global Health Estimates. https://www.who.int/data/global-health-estimates (2024).

[5] Tonelli M, Nangaku M, Machalska M et al. Guatemala’s resolution on kidney health: a historic opportunity. Lancet 2025;405:1809–10. 10.1016/S0140-6736(25)00657-9 40373784

[6] KDIGO; KDIGO 2024 Clinical Practice Guideline for the evaluation and management of chronic kidney disease. Kidney Int 2024;105:S117–s314. 38490803 10.1016/j.kint.2023.10.018

[7] NICE. Chronic Kidney Disease. NICE Clinical Knowledge Summary https://cks.nice.org.uk/topics/chronic-kidney-disease/ (last revised May 2025; date last accessed July 7th 2025).

[8] Neuen B, Green J, Vaduganathan M.. Improving outcomes in cardio-renal-metabolic diseases. J Card Fail 2022;28:1573–4. 36368805 10.1016/j.cardfail.2022.10.421

[9] Rangaswami J, Tuttle K, Vaduganathan M.; Cardio-renal-metabolic care models: toward achieving effective interdisciplinary care. Circ: Cardiovasc Qual Outcomes 2020;13:e007264. 33176463 10.1161/CIRCOUTCOMES.120.007264 PMC7673632

[10] Chatzis DG, Kolokathis K, Magounaki K et al. Changing the concept: from the traditional glucose-centric to the new cardiorenal-metabolic approach for the treatment of type 2 diabetes. touchREV Endocrinol 2021;17:92. 10.17925/EE.2021.17.2.92 35118454 PMC8676106

[11] Sebastian SA, Padda I, Johal G.; Cardiovascular-kidney-metabolic (CKM) syndrome: a state-of-the-art review. Curr Probl Cardiol 2024;49:102344. 10.1016/j.cpcardiol.2023.102344 38103820

[12] Jankowski J, Floege J, Fliser D et al. Cardiovascular disease in chronic kidney disease: pathophysiological insights and therapeutic options. Circulation 2021;143:1157–72. 10.1161/CIRCULATIONAHA.120.050686 33720773 PMC7969169

[13] Zoccali C, Mallamaci F, Adamczak M et al. Cardiovascular complications in chronic kidney disease: a review from the European Renal and Cardiovascular Medicine Working Group of the European Renal Association. Cardiovasc Res 2023;119:2017–32. 10.1093/cvr/cvad083 37249051 PMC10478756

[14] Smart NA, Dieberg G, Ladhani M et al. Early referral to specialist nephrology services for preventing the progression to end-stage kidney disease. Cochrane Database Syst Rev 2014;6:CD007333. 10.1002/14651858.CD007333.pub2 24938824

[15] United States Renal Data System. *USRDS Annual Data Report: Epidemiology of Kidney Disease in the United States*. https://www.niddk.nih.gov/about-niddk/strategic-plans-reports/usrds/news/2023/usrds-releases-2023-interactive-annual-data-report (12 July 2025, date last accessed).

[16] UK Renal Registry. UK Renal Registry 26th Annual Report. UK Renal Registry, https://ukkidney.org/audit-research/annual-report 2024. (12 July 2025, date last accessed).

[17] CVDPREVENT. Cardiovascular Disease Prevention Audit. For the audit period up to June 2024. CVDPREVENT, https://www.cvdprevent.nhs.uk/2024 (12 July 2025, date last accessed).

[18] Hirst JA, Hill N, O’Callaghan CA et al. Prevalence of chronic kidney disease in the community using data from OxRen: a UK population-based cohort study. Br J Gen Pract 2020;70:e285–93. 10.3399/bjgp20X708245 (12 July 2025, date last accessed). 32041766 PMC7015167

[19] Parving HH, Lewis JB, Ravid M et al. Prevalence and risk factors for microalbuminuria in a referred cohort of type II diabetic patients: a global perspective. Kidney Int 2006;69:2057–63. 10.1038/sj.ki.5000377 16612330

[20] Public Health England. INLIQ (indicators no longer in QOF) information 2018/19. https://fingertips.phe.org.uk/documents/INLIQ_2018.19_summary_final.pdf: 2018/2019 (12 July 2025, date last accessed).

[21] Stewart S, Kalra PA, Blakeman T et al. Chronic kidney disease: detect, diagnose, disclose—a UK primary care perspective of barriers and enablers to effective kidney care. BMC Med 2024;22:331. 10.1186/s12916-024-03555-0 39148079 PMC11328380

[22] NHS Digital. National Diabetes Audit 2021-2022 NHS. https://digital.nhs.uk/data-and-information/publications/statistical/national-diabetes-audit-type-1-diabetes 2023. (12 July 2025, date last accessed).

[23] Baig A, Zafar A. Urine ACR uptake in patients with a diagnosis of type 1 and 2 diabetes mellitus in a primary care setting: a cross sectional study. Primary Care Diabetes 2023;17:639–42. 10.1016/j.pcd.2023.10.005 37839987

[24] Winocour P, Diggle J, Davies S et al. Testing for kidney disease in type 2 diabetes: consensus statement and recommendations. Diabetes Primary Care 2020;22:99–109.

[25] Tangri N, Peach EJ, Franzén S et al. Patient management and clinical outcomes associated with a recorded diagnosis of stage 3 chronic kidney disease: The REVEAL-CKD Study. Adv Ther 2023;40:2869–85. 10.1007/s12325-023-02482-5 37133647 PMC10219868

[26] Kidney Care Kidney Care UK. Let’s talk kidneys: opportunities for early intervention in chronic kidney disease. Kidney Care UK, Policy Report. 2023; https://kcuk.cdn.ngo/media/documents/Kidney_Care_UK_-_CKD_Lets_talk_kidneys_report_-_Hyperlinks.pdf (24 November 2025, date last accessed).

[27] Thomas N, Ewart C, Hill C. Evaluating the feasibility and acceptability of home-based urinalysis for albumin-creatinine ratio with smartphone technology: a quality improvement project. J Renal Care 2024;50:104–11. 10.1111/jorc.12460 36786046

[28] The Dutch Cardiovascular Alliance. Check@Home. https://dcvalliance.nl/our-consortia/checkhome/ (2022). (9 July 2025, date last accessed).

[29] Van Mil D, Kieneker LM, Evers-Roeten B et al. Participation rate and yield of two home-based screening methods to detect increased albuminuria in the general population in the Netherlands (THOMAS): a prospective, randomised, open-label implementation study. Lancet 2023;402:1052–64. 10.1016/S0140-6736(23)00876-0 37597522

[30] Pouwels XG, Van Mil D, Kieneker LM et al. Cost-effectiveness of home-based screening of the general population for albuminuria to prevent progression of cardiovascular and kidney disease. EClinlMed 2024;68. 10.1016/j.eclinm.2023.102414 PMC10827681 38299045

[31] Al-Chalabi S, Alderson H, Garratt N et al. Improving outpatient clinic experience: the future of chronic kidney disease care and associated multimorbidity. BMJ Open Qual 2023; 12 e002188. 10.1136/bmjoq-2022-002188 PMC10401237 37532458

[32] Renal Service Transformation Programme. Renal Toolkit. NHS, https://future.nhs.uk/RSTP/view?objectID=33736176 2023; (9 July 2025, date last accessed).

[33] Major RW, Lakhani N, Ahmed Y et al. Integrated primary and secondary care optimises the management of people with CKD-The LUCID project. Clin Kidney J 2025;18: , sfaf049. 10.1093/ckj/sfaf049 40207101 PMC11980979

[34] Helminski D, Sussman JB, Pfeiffer PN et al. Development, implementation, and evaluation methods for dashboards in health care: scoping review. JMIR Med Inform 2024;12:e59828. 10.2196/59828 39656991 PMC11651422

[35] Dowding D, Randell R, Gardner P et al. Dashboards for improving patient care: review of the literature. Int J Med Informatics 2015;84:87–100. 10.1016/j.ijmedinf.2014.10.001 25453274

[36] Dagliati A, Sacchi L, Tibollo V et al. A dashboard-based system for supporting diabetes care. J Am Med Inform Assoc 2018;25:538–47. 10.1093/jamia/ocx159 29409033 PMC7647008

[37] Rea RD, Lumb A, Tan GD et al. Using data to improve the care of people with diabetes across Oxfordshire. Practical Diabetes 2020;37:27–31. 10.1002/pdi.2257

[38] Yakovchenko V, Jacob DA, Rogal SS et al. User experience of a hepatitis C population management dashboard in the Department of Veterans Affairs. PLoS ONE 2023;18:e0285044. 10.1371/journal.pone.0285044 37130107 PMC10153746

[39] Samadbeik M, Engstrom T, Lobo EH et al. Healthcare dashboard technologies and data visualization for lipid management: a scoping review. BMC Med Inform Decis Mak 2024;24:352. 10.1186/s12911-024-02730-w 39574106 PMC11583543

[40] Khairat SS, Dukkipati A, Lauria HA et al. The impact of visualization dashboards on quality of care and clinician satisfaction: integrative literature review. JMIR Hum Factors 2018;5:e22. 10.2196/humanfactors.9328 29853440 PMC6002673

[41] Zhuang M, Concannon D, Manley E. A framework for evaluating dashboards in healthcare. IEEE Trans Visual Comput Graphics 2022;28:1715–31. 10.1109/TVCG.2022.3147154 35213306

[42] Tangri N, Stevens LA, Griffith J et al. A predictive model for progression of chronic kidney disease to kidney failure. JAMA 2011;305:1553–9. 10.1001/jama.2011.451 21482743

[43] Major RW, Shepherd D, Medcalf JF et al. The Kidney Failure Risk Equation for prediction of end stage renal disease in UK primary care: an external validation and clinical impact projection cohort study. PLoS Med 2019;16:e1002955. 10.1371/journal.pmed.1002955 31693662 PMC6834237

[44] Imison C, Curry N, Holder H et al. Shifting the balance of care: great expectations. Nuffield Trust 2017;.

[45] Reynolds R, Dennis S, Hasan I et al. A systematic review of chronic disease management interventions in primary care. BMC Fam Pract 2018;19:1–13. 10.1186/s12875-017-0692-3 29316889 PMC5759778

[46] Zhang Y, He D, Zhang W et al. ACE inhibitor benefit to kidney and cardiovascular outcomes for patients with non-dialysis chronic kidney disease stages 3–5: a network meta-analysis of randomised clinical trials. Drugs 2020;80:797–811. 10.1007/s40265-020-01290-3 32333236 PMC7242277

[47] EMPA-Kidney Collaborative Group. Empagliflozin in patients with chronic kidney disease. N Engl J Med 2023;388:117–27. 10.1056/NEJMoa2204233 36331190 PMC7614055

[48] Heerspink HJ, Stefánsson BV, Correa-Rotter R et al. Dapagliflozin in patients with chronic kidney disease. N Engl J Med 2020;383:1436–46. 10.1056/NEJMoa2024816 32970396

[49] Bakris GL, Agarwal R, Anker SD et al. Effect of finerenone on chronic kidney disease outcomes in type 2 diabetes. N Engl J Med 2020;383:2219–29. 10.1056/NEJMoa2025845 33264825

[50] Forbes AK, Hinton W, Feher MD et al. Implementation of chronic kidney disease guidelines for sodium-glucose co-transporter-2 inhibitor use in primary care in the UK: a cross-sectional study. EClinMed 2024;68:102426. 10.1016/j.eclinm.2024.102426 PMC10831804 38304744

[51] Eberly LA et al. Association of race/ethnicity, gender, and socioeconomic status with sodium-glucose cotransporter 2 inhibitor use among patients with diabetes in the United States. JAMA Netw Open 2021;4:e2139152. 10.1001/jamanetworkopen.2021.6139 PMC8050743 33856475

[52] NICE Finerenone for treating chronic kidney disease in type 2 diabetes. NICE, https://www.nice.org.uk/guidance/ta877/resources/finerenone-for-treating-chronic-kidney-disease-in-type-2-diabetes-pdf-82613678191045: 2023; (9 July 2025, date last accessed). 40112124

[53] Nuffield Department of Primary Care Health Sciences. *https://openprescribing.net/*. https://openprescribing.net/ 2025; (9 July 2025, date last accessed).

[54] Perkovic V, Tuttle KR, Rossing P et al. Effects of semaglutide on chronic kidney disease in patients with type 2 diabetes. N Engl J Med 2024;391:109–21. 10.1056/NEJMoa2403347 38785209

[55] England; NHS. Interim commissioning guidance—implementation of the NICE Technology Appraisal TA1026 and the NICE funding variation for tirzepatide (Mounjaro) for the management of obesity. NHS England 2025; PRN01879. https://www.england.nhs.uk/publication/interim-commissioning-guidance-implementation-of-the-nice-technology-appraisal-ta1026-and-the-nice-funding-variation-for-tirzepatide-mounjaro-for-the-management-of-obesity/ (24 November 2025, date last accessed).

[56] Santschi V, Chiolero A, Burnand B et al. Impact of pharmacist care in the management of cardiovascular disease risk factors: a systematic review and meta-analysis of randomized trials. Arch Intern Med 2011;171:1441–53. 10.1001/archinternmed.2011.399 21911628

[57] NHS. The NHS Long Term Plan. National Health Service: 2019.

[58] Hull S, Rajabzadeh V, Thomas N et al. Do virtual renal clinics improve access to kidney care? A preliminary impact evaluation of a virtual clinic in East London. BMC Nephrol 2020;21:1–9. 10.1186/s12882-020-1682-6 PMC6954525 31924178

[59] Basudev N, Crosby-Nwaobi R, Thomas S et al. A prospective randomized controlled study of a virtual clinic integrating primary and specialist care for patients with type 2 diabetes mellitus. Diabet Med 2016;33:768–76. 10.1111/dme.12985 27194175

[60] Harnett P, Jones M, Almond M et al. A virtual clinic to improve long-term outcomes in chronic kidney disease. Clin. Med 2018;18:356–63. 10.7861/clinmedicine.18-5-356 PMC6334099 30287426

[61] Mark D, Fitzmaurice G, Haughey K et al. Assessment of the quality of care and financial impact of a virtual renal clinic compared with the traditional outpatient service model. Int J Clin Pract 2011;65:1100–7. 10.1111/j.1742-1241.2011.02750.x 21923849

[62] Katz IJ, Pirabhahar S, Williamson P et al. iConnect CKD–virtual medical consulting: a web-based chronic kidney disease, hypertension and diabetes integrated care program. Nephrology 2018;23:646–52. 10.1111/nep.13070 28474361

[63] Major RW, Brown C, Shepherd D et al. The Primary-Secondary Care Partnership to Improve Outcomes in Chronic Kidney Disease (PSP-CKD) Study: a cluster randomized trial in primary care. J Am Soc Nephrol 2019;30:1261–70. 10.1681/ASN.2018101042 31097609 PMC6622412

[64] Jhamb M, Weltman MR, Devaraj SM et al. Electronic health record population health management for chronic kidney disease care: a cluster randomized clinical trial. JAMA Intern. Med. 2024;184:737–47. 10.1001/jamainternmed.2024.0708 38619824 PMC11019443

[65] Jhamb M, Weltman MR, Yabes JG et al. Electronic health record based population health management to optimize care in CKD: design of the kidney coordinated health management partnership (K-CHAMP) trial. Contemp Clin Trials 2023;131:107269. 10.1016/j.cct.2023.107269 37348600 PMC10529809

[66] Weltman MR, Lavenburg L-MU, Han Z et al. Population health management and guideline-concordant care in CKD: a secondary analysis of K-CHAMP. J Am Soc Nephrol 2024;10.1681. 10.1681/ASN.0000000544 PMC12059108 39485493

[67] Liu P, Sawhney S, Heide-Jørgensen U et al. Predicting the risks of kidney failure and death in adults with moderate to severe chronic kidney disease: multinational, longitudinal, population based, cohort study. BMJ 2024;385:e078063. 10.1136/bmj-2023-078063 38621801 PMC11017135

[68] Thanabalasingam SJ, Iliescu EA, Norman PA et al. Kidney failure risk equation thresholds for multidisciplinary kidney care referrals: a validation study. Kidney Medicine 2024;6:100805. 10.1016/j.xkme.2024.100805 38562968 PMC10982608

[69] Donald M, Weaver RG, Smekal M et al. Implementing a formalized risk-based approach to determine candidacy for multidisciplinary CKD Care: a descriptive cohort study. Can J Kidney Health Dis 2023;10:20543581231215865. 10.1177/20543581231215865 38044897 PMC10693221

[70] Hahn Lundström U, Ramspek CL, Dekker FW et al. Clinical impact of the Kidney Failure Risk Equation for vascular access planning. Nephrol Dial Transplant 2024;39:2079–87. 10.1093/ndt/gfae064 38486367 PMC11648961

[71] Atiquzzaman M, Zhu B, Romann A et al. Kidney Failure Risk Equation in vascular access planning: a population-based study supporting value in decision making. Clin Kidney J 2024;17:sfae008. 10.1093/ckj/sfae008 38327282 PMC10847629

[72] NHS Digital. National Diabetes Audit: report 1–care processes and treatment targets. NHS Information Centre., https://digital.nhs.uk/services/national-diabetes-audit: 2011–22. (9 July 2025, date last accessed).

[73] Tuot DS, Plantinga LC, C-y H et al. Chronic kidney disease awareness among individuals with clinical markers of kidney dysfunction. Clin J Am Soc Nephrol 2011;6:1838–44. 10.2215/CJN.00730111 21784832 PMC3156423

[74] Devins GM, Mandin H, Hons RB et al. Illness intrusiveness and quality of life in end-stage renal disease: comparison and stability across treatment modalities. Health Psychol 1990;9:117. 10.1037/0278-6133.9.2.117 2331973

[75] Narva AS, Norton JM, Boulware LE. Educating patients about CKD: the path to self-management and patient-centered care. Clin J Am Soc Nephrol 2016;11:694–703. 10.2215/CJN.07680715 26536899 PMC4822666

[76] Fraser SD, Blakeman T. Chronic kidney disease: identification and management in primary care. POR 2016;7:21–32. 10.2147/POR.S97310 PMC5087766 27822135

[77] Allen AS, Forman JP, Orav EJ et al. Primary care management of chronic kidney disease. J Gen Intern Med 2011;26:386–92. 10.1007/s11606-010-1523-6 20922494 PMC3055964

[78] Blakeman T, Protheroe J, Chew-Graham C et al. Understanding the management of early-stage chronic kidney disease in primary care: a qualitative study. Br J Gen Pract 2012;62:e233–42. 10.3399/bjgp12X636056 22520910 PMC3310029

